# Antigen‐presenting cells maintain T cells proliferation through vesicle transfer of telomeres: A new approach to telomere extension

**DOI:** 10.1002/mco2.270

**Published:** 2023-06-03

**Authors:** Xiaorui Zhang, Guoqing Tong

**Affiliations:** ^1^ Department of Reproductive Medicine The First Affiliated Hospital of Xi'an Jiaotong University Shaanxi People's Republic of China

1

Recently, a study was published in *Nature Cell Biology* reporting a novel immune cell repair mechanism, namely, “intercellular transfer of telomeres”. Lanna et al.^1^ found that telomeres can be transferred from antigen‐presenting cells (APCs) to T cells, rejuvenating senescent T cells and promoting the formation of long‐term immune memory. This study has significant implications for understanding the mechanisms of aging, maintaining immune system health, and finding new ways to treat age‐related diseases.

Telomeres are tandem repeats of (TTAGGG) nucleotides located at the ends of chromosomes that play a crucial role in maintaining chromosomal stability during cell division. Usually, telomeres are positioned in a chromosomally dense structure consisting of telomeric DNA and six protein complexes including TRF1, TRF2, TIN2, TPP1, RAP1, and POT1, these six proteins together form the shelterin structure and maintain telomere homeostasis.^2^ However, with each successive cell division, telomeres shorten, eventually reaching a critically short length that triggers replicative senescence, also known as the Hayflick limit. In this state, cells cease to divide, leading to a loss of proliferative activity.

To prevent cells from entering an irreversible process due to replicative senescence, a small proportion of cell types, including lymphocytes (T‐ and B‐cells), germ line cells, stem cells and tumor cells have a telomere maintenance mechanism. This is achieved through two main routes: the telomerase pathway and the alternative lengthening of telomeres (ALT) pathway. Telomerase is a reverse transcriptase that increases telomere length by directly adding telomeric fragments to the 3′ ends of chromosomes. In addition to the telomerase pathway, a non‐telomerase‐dependent telomere lengthening pathway‐ALT, which is achieved by homologous recombination (HR) and more frequent telomeric sister chromatin exchange.^3^ The two telomere extension modalities can function independently to ultimately lengthen telomeres. T cells maintain their telomere length mainly through the telomerase pathway. The activity of telomerase in T cells is dependent on T‐cell subtypes, status, and age. In young and healthy individuals, T cells exhibit relatively high levels of telomerase activity, whereas in elderly and immunocompromised individuals, telomerase activity in T cells decreases.^4^

During an infection, APCs such as dendritic cells and macrophages present exogenous antigen fragments (such as pathogen proteins or intracellular proteins) to T‐cell receptors, leading to the activation of specific T‐cell types.^4^ After the pathogen has been cleared, the majority of T cells undergo cell death, while a small proportion differentiates into memory T cells, which have a higher antigen recognition capacity, faster response time, and longer survival compared to naïve T cells.^4^ APCs present antigens to T cells via synapses, and this cell‐to‐cell communication occurs during the initial immunization and re‐immunization processes. T cells remain in a resting or quiescent state in the absence of stimulation, exhibiting little or no activity. Upon activation by antigens, T cells rapidly divide and proliferate, while also activating telomerase to maintain their telomere length and avoid telomere attrition due to proliferation. However, the activation of low levels of telomerase is insufficient to prevent T cells from entering a state of proliferative exhaustion, which results in senescence^4^ (Figure 1A). This presents a paradox, as T cells are known for their long‐lasting immune function. Therefore, it is likely that there are other mechanisms by which T cells can maintain telomeres and evade senescence.

**FIGURE 1:**
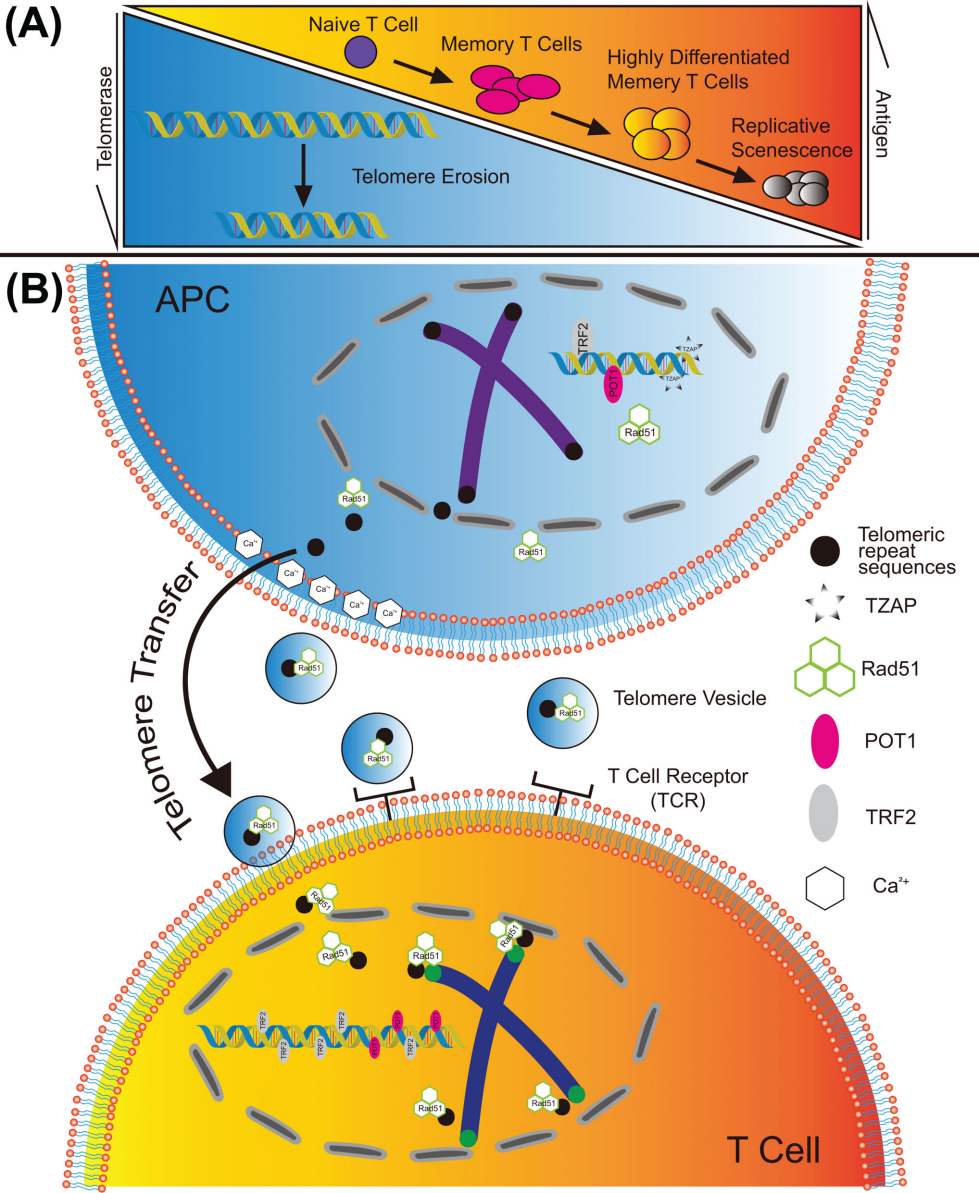
(A) T‐cell senescence processes and a new approach to telomere extension. Differentiated T cells have progressively lower telomerase activity and shorter telomere length. (B) Antigen‐presenting cells (APCs) maintain T‐cell proliferation through the vesicle transfer of telomeres. After calcium (Ca^2+^) signaling activation and synapse formation events, there is a degradation of shelterin proteins, leading to the accumulation of telomeric zinc finger‐associated protein (TZAP). This accumulation subsequently leads to the generation of telomeric repeat sequences, which are packaged into vesicles with Rad51 and delivered to T cells upon T cell receptor/major histocompatibility complex class II (TCR/MHC‐II) activation. The homologous recombination process, facilitated by Rad51, ultimately results in the extension of telomeres in T cells.

Lanna et al. demonstrated that certain T cells, predominantly naïve and central memory cells, utilize alternative mechanisms for telomere elongation, in addition to telomerase.^1^ In the presence of APCs, CD27^+^ CD28^+^ CD4^+^ CD3^+^ T cells can elongate telomeres by approximately 3 kb. As lymphocytes and APCs form antigen‐specific connections and immune synapses, T cells may receive antigenic stimulation while simultaneously acquiring telomeres from APCs.^1^ Importantly, it was observed that telomerase‐deficient T cells (TERT‐knockout), generated via CRISPR/Cas9, underwent telomere elongation even when exposed to APCs and antigens, indicating the existence of an alternative, non‐telomerase‐dependent pathway for T cell telomere extension. When T cells receive antigenic stimulation, they produce significant quantities of telomerase.^1^ However, telomerase activity in T cells declines following the first time encounter with an antigen, resulting in an inability to maintain telomere length and inducing senescence, ultimately impairing immune system function.^4^

To confirm that the telomeres elongated in T cells originated from APCs, the scientists used telomere FISH and were able to observe telomeres exiting APCs and entering APC‐T cell synapses.^1^ The vesicle is rapidly released upon stimulation with ionomycin treatment. Upon activation by binding to T cells, the telomeres of APCs were transferred to the nuclei of T cells via vesicle fractions. The researchers observed that EdU‐labeled APC telomeres co‐localized with FISH‐labeled T‐cell telomeres and EdU‐labeled telomeric DNA donated by APCs was detected in approximately 8% of metaphase T‐cell chromosome ends.^1^ This indicates that a second telomere lengthening pathway exists in some T cells.

Specific prerequisites must be met for this transfer process to occur. Lanna et al. found that APCs, ionomycin activated or in synapse with T cells result in the down‐regulation of the expression of important factors involved in telomere maintenance and protection, including POT1 and telomeric repeat binding factor 2 (TRF2).^1^ These proteins take part in the DNA damage response by consolidating at telomeres and protecting their stability. And the calcium ionophore ionomycin stimulates the accumulation of telomeric zinc finger‐associated protein (TZAP) in vesicles. Telomere transfer is prevented by shelterin proteins, and the degradation of these proteins can result in the release of telomeres in extracellular vesicles through the action of TZAP, which causes telomere excision.^5^ Therefore, the accumulation of TZAP and degradation of shelterin proteins are the primary conditions that allow for telomere transfer. The authors also discovered that Rad51, a critical protein involved in telomere elongation through HR, was present in telomere vesicles.^1^ Knockdown of Rad51 resulted in the inability of APCs to translocate telomeres into T cells, leading to shortened telomere lengths in T cells.^1^

When telomeres are transferred to vesicles, the transport of vesicles between APCs and T cells becomes critical. The scientists discovered that antigen‐specific contacts through the T cell receptor (TCR) and major histocompatibility complex class II (MHC‐II) play a critical role in the initiation of telomere transfer.^1^ This process stimulates calcium flux in APCs, and the administration of calcium flux inhibitors considerably attenuates the telomere transfer process in APCs. It should be noted that MHC signaling plays an essential role in initiating telomere transfer during antigen‐specific TCR recognition under physiological conditions.^1^ However, it is not required for the fusion of T‐cell telomeres after the transfer. After the transfer of telomere vesicles, Rad51 was responsible for extending T‐cell telomeres through recombination. Although the removal of Rad51 did not affect the number or size of the vesicles, it significantly impaired the extension of T‐cell telomeres.^1^

Therefore, successful translocation of telomeres from APCs to T cells requires a coordinated process that involves TCR/MHC‐II activation, TZAP accumulation, degradation of shelterin proteins, and the presence of Rad51 within the vesicles (Figure 1B). To examine the effect of this telomere lengthening on T cells, it was found that T cells receiving vesicular telomere transport had a nearly 3‐fold increase in proliferation rate over 30–40 days compared to T cells without vesicular transport telomeres and that this increase also maintained the ability of T cells to generate immune memory and show prolonged immune resistance to an influenza virus in mice.^1^

Understanding this new process can provide novel insights for prospective long‐term immunotherapeutic strategies. The newly discovered process has the potential to facilitate telomere extension in selected T cell types, like memory T cells, that experience telomerase depletion, thereby enabling the preservation of immune function. However, the mechanisms that underlie the differential ability of T cells to undergo this mode of telomere extension and its relationship with telomerase activity necessitate further investigation.

## AUTHOR CONTRIBUTIONS

Xiaorui Zhang conceived and drafted the manuscript. Xiaorui Zhang drew the figures. Guoqing Tong provided valuable discussion and revised the manuscript. Both authors have read and approved the final manuscript.

## CONFLICT OF INTEREST STATEMENT

The authors declare no conflict of interest.

## FUNDING INFORMATION

Not applicable.

## ETHICS STATEMENT

Not applicable.

## Data Availability

Not applicable.
